# Similar cognitive characteristics between gaming disorder and other psychiatric disorders

**DOI:** 10.1192/j.eurpsy.2024.667

**Published:** 2024-08-27

**Authors:** S. Tei, J. Fujino

**Affiliations:** ^1^Department of Psychiatry, Kyoto University, Kyoto; ^2^Department of Psychiatry and Behavioral Sciences, Tokyo Medical and Dental University, Tokyo, Japan

## Abstract

**Introduction:**

Cognitive characteristics that differentiate normal from problematic gaming need to be identified, owing to the growing popularity of internet games and the rapid rise in mental health problems. Gaming disorder (GD) involves playing games despite their negative effects and is often related to unsuccessful attempts to reduce gaming. GD frequently results in adverse outcomes related to education, employment, and social responsibilities, thereby significantly influencing daily life.

**Objectives:**

We aimed to elucidate the neurocognitive features underlying GD development and preservation, and possible overlapping features between GD and other psychiatric disorders.

**Methods:**

We performed a literature search to identify GD-related studies. We focused on two key aspects: (a) altered executive functions (EFs) and (b) gaming urge. We mainly searched the PubMed and Web of Science databases using relevant keywords. All retrieved literature were assessed for eligibility to reduce selection biases.

**Results:**

Our preliminary review identified that GD features prominent deficits in EFs, including cognitive inflexibility, poor response inhibition, altered decision-making, and intensified susceptibility to game-related stimuli. These deficits were found to be associated with abnormal neural activity in brain regions subserving EFs and reward-based learning. Hence, excessive gaming may maladaptively suppress controlled and conscious processing, which can amplify automatic and implicit processes to develop gaming urges. In addition, many of these neuropsychological deficits have been observed in other addictions and seemingly unrelated disorders such as autism spectrum disorder (ASD). Similar EF deficits have been identified in ASD, which involve reduced cognitive flexibility and related dysfunction, including excessive attention focus, restricted interest, maladaptive reward processing, and reduced self-control. However, there is considerable variation among individuals and study methods, which requires more comprehensive research strategies.

**Conclusions:**

We elucidated comparable cognitive features among individuals with GD, addiction disorders, and ASD. These similarities provide clues regarding GD etiology, ideas for improving preventative therapies, and markers for risk evaluation. Additional investigations on how GD and other disorders possess similar and distinctive cognitive functions are worth pursuing. It is also crucial to further examine the extent of shared cognitive features in the general population, wherein the peripheral pathological characteristics lie on a continuum with typical and atypical populations.

**Disclosure of Interest:**

None Declared

